# The BiZiFED project: Biofortified zinc flour to eliminate deficiency in Pakistan

**DOI:** 10.1111/nbu.12362

**Published:** 2019-01-17

**Authors:** H. Ohly, M. R. Broadley, E. J. M. Joy, M. J. Khan, H. McArdle, M. Zaman, M. Zia, N. Lowe

**Affiliations:** ^1^ University of Central Lancashire Preston UK; ^2^ University of Nottingham Nottingham UK; ^3^ London School of Hygiene & Tropical Medicine London UK; ^4^ Khyber Medical University Peshawar Pakistan; ^5^ Khyber Medical College and Teaching Hospital Peshawar Pakistan; ^6^ Fauji Fertilizer Company Limited Rawalpindi Pakistan

**Keywords:** biofortification, deficiency, micronutrients, Pakistan, zinc

## Abstract

Zinc deficiency is a global public health problem, affecting ~17% of the world's population, with the greatest burden in low‐ and middle‐income countries. An increasing body of evidence suggests that biofortification may be a cost‐effective and sustainable approach to reducing zinc and other micronutrient deficiencies. Biofortification enhances the nutritional quality of food crops through conventional plant breeding techniques and agronomic practices. This paper presents ongoing research on biofortification in Pakistan, where over 40% of women are zinc deficient. The *Biofortified Zinc Flour to Eliminate Deficiency* (*BiZiFED*) project aims to investigate the impact of biofortification as a strategy to alleviate zinc deficiency in Pakistan. The project is supported by the Biotechnology and Biological Sciences Research Council (BBSRC) Global Challenges Research Fund from May 2017 to April 2019. This paper outlines the four objectives and work packages within the *BiZiFED* project: (1) a double‐blind, randomised controlled trial to examine the effect of consuming flour made from a high zinc variety of biofortified wheat (Zincol‐2016/NR‐421) on dietary zinc intake and status; (2) a cost‐effectiveness study to assess the health and economic impact of agronomic biofortification of wheat; (3) a mixed methods study to explore the cultural acceptability and sustainability of biofortification in Pakistan; (4) capacity building and development of long‐term research partnerships in Pakistan. The findings will contribute to the evidence base for the potential impact of biofortification to alleviate zinc deficiency among the poorest communities.

## Introduction

Zinc deficiency is a global public health problem, affecting ~17% of the world's population, with the greatest burden in low‐ and middle‐income countries (LMIC) (Wessells & Brown 2012; Kumssa *et al*. 2015). The most recent *National Nutrition Survey* in Pakistan indicated that over 40% of women were zinc deficient (Bhutta *et al*. 2011). The health consequences of zinc deficiency include stunted growth and impaired neurodevelopment in children, increased susceptibility to infections in children and adults, and complications during pregnancy and childbirth (Brown *et al*. 2004). Therefore, it is imperative for global health and development that sustainable, cost‐effective solutions to zinc deficiency are found.

Attempts to alleviate zinc deficiency (and other micronutrient deficiencies) in LMIC have followed several strategies, including supplementation and food fortification. While these strategies have demonstrated positive outcomes on health and wellbeing, such as improved child growth and nutritional status associated with multiple micronutrient supplementation (Allen *et al*. 2009), programmes may be resource intensive and difficult to implement and sustain. Furthermore, they often exclude hard to reach rural communities, where most food is grown and processed locally. Over 60% of Pakistan's wheat harvest is retained by farmers for self‐consumption or used as in‐kind payments to labourers (Ansari *et al*. 2018). Most of this wheat is milled to flour locally and not in the large industrial mills where flour fortification occurs.

An increasing body of evidence suggests that biofortification – a process by which the nutritional quality of food crops is improved through plant breeding and agronomic practices – may be a cost‐effective and sustainable approach to reducing micronutrient deficiencies, which complements other strategies such as supplementation and food fortification (Bouis & Saltzman 2017; Lockyer *et al*. 2018).

Tightly controlled human feeding studies (efficacy trials) have demonstrated that consumption of biofortified crops can lead to increased micronutrient status (Saltzman *et al*. 2017). A limited number of effectiveness studies have demonstrated similar improvements under ‘real world’ conditions. Furthermore, most studies to date have tested crops biofortified with vitamin A and iron. Less is known about the efficacy and effectiveness of crops biofortified with zinc, which were released into target populations more recently.

Another key challenge is to assess the impact of biofortification in the context of spatially variable soils, with different concentrations of plant‐available zinc, differences in basic soil properties that affect zinc uptake, and subject to differing management strategies given the variations in resource endowment of farmers and in their physical environment (Joy *et al*. 2015a,b; Manzeke *et al*. 2017).

The *Biofortified Zinc Flour to Eliminate Deficiency* (*BiZiFED*) project aims to investigate the impact of biofortification as a strategy to alleviate zinc deficiency in Pakistan. The project is supported by the Biotechnology and Biological Sciences Research Council (BBSRC) Global Challenges Research Fund from May 2017 to April 2019. This paper outlines the four objectives and work packages within the *BiZiFED* project. Our findings will be published in 2019.

## Examining the effectiveness of biofortified flour as a strategy to alleviate zinc deficiency in women of child‐bearing age in low‐resource settings in Pakistan

Objective 1 of the *BiZiFED* project is to examine the effect of consuming flour made from a high zinc variety of biofortified wheat (Zincol‐2016/NR‐421) on dietary zinc intake and status, using established and novel biomarkers of zinc status that have potential for use in low‐resource settings. A double‐blind, randomised controlled trial (RCT) was conducted in a rural brick kiln community in Peshawar District, Khyber Pakhtunkhwa Province, North West Pakistan (Lowe *et al*. 2018). This is a poor and marginalised community with low dietary diversity. Wheat flour chapattis are traditionally consumed with every meal. We worked in partnership with Khyber Medical University (KMU) and the Abaseen Foundation (a UK/Pakistan charity) to plan and deliver the RCT.

Fifty households were recruited in September 2017. Each household included a female aged 16–49 years, who was neither pregnant nor breastfeeding, as the primary study participant. Households were provided with flour milled from biofortified wheat grain (Zincol‐2016/NR‐421, grown with zinc fertilisers) or control wheat grain (Galaxy‐2013). All households received control flour for an initial 2‐week baseline period. In the first 8‐week intervention period, 25 households received biofortified flour (Group A) and 25 households received control flour (Group B). In the second 8‐week intervention period, Group A and B crossed over and received control flour and biofortified flour, respectively. Tissue samples (blood, hair and nails) were collected from the women at five time‐points: baseline, mid and end of period 1, and mid and end of period 2. This study will compare established biomarkers of zinc status (plasma zinc concentration) with novel indicators, including markers of DNA damage (Zyba *et al*. 2017) and a new laser technique for measuring nail and hair zinc concentration. The trial protocol was prospectively registered (ISRCTN83678069) and published (Lowe *et al*. 2018).

The RCT was successfully completed in February 2018. We achieved 90% retention due to effective community engagement by our research partners. Baseline analysis confirmed that 52% of female participants were zinc deficient, defined as plasma zinc concentration less than 700 μg/l. At the time of submission of this manuscript in November 2018, tissue samples were being analysed in specialist laboratories in Pakistan, the UK and the US.

## Investigating the potential health and economic impact of agronomic biofortification of wheat in Pakistan

Objective 2 of the *BiZiFED* project is to quantify the health and economic impact of the high zinc wheat variety (Zincol‐2016/NR‐421) grown at sites of contrasting available soil zinc status and under different zinc fertiliser regimes. Zinc fertilisation can increase yield and enhance zinc concentration in the edible part of the wheat grain (Joy *et al*. 2015a,2015b, 2017; Cakmak & Kutman 2017). Our study will evaluate the cost‐effectiveness of genetic (breeding) and agronomic (fertiliser) biofortification.

Field experiments were conducted in partnership with Fauji Fertilizer Company, the University of Agriculture Faisalabad (UAF), the National Agriculture Research Centre (NARC) and the Cereal Crops Research Institute (CCRI). Three sites in Pakistan were selected based on the contrasting plant‐available zinc status of their soils – high, medium and low. Replicated plots of biofortified wheat (Zincol‐2016/NR‐241) and local reference varieties of wheat were sown at each site in November 2017, using appropriate randomised designs. Eight different zinc fertiliser treatments were applied to the wheat crop, including basal applications (to the soil), foliar applications (to the leaves) in various concentrations and combinations of both at various growth stages. Crop traits were recorded at maturity from randomly selected plants at each site, including plant height, number of tillers per square metre, spike length, number of grains per spike and weight of grains per spike.

The wheat crop was harvested at all three sites in May 2018. The grain was manually separated, threshed, cleaned and weighed. The grains have been analysed for mineral composition. Data are currently being analysed to determine the effects of various treatments, either applied alone or in combination, on the grain yield and zinc concentration of the wheat grain. The health and economic impact of genetic and agronomic biofortification will be calculated in terms of disability‐adjusted life years (DALYs), which is likely to be substantial for Pakistan (Joy *et al*. 2017). Results are expected in March 2019.

## Exploring the cultural acceptability and sustainability of biofortification in Pakistan

Objective 3 of the *BiZiFED* project is to explore the cultural context, traditions, knowledge and attitudes of local stakeholders to genetic biofortification and the use of zinc fertilisers to enhance dietary zinc intake and increase crop yield. Stakeholder mapping activities continued throughout the 2‐year study duration. The *BiZiFED* project team (and selected local stakeholders) contributed through email consultations, interactive workshops and discussions. A comprehensive list of stakeholders was developed, from wheat producers to flour consumers. This evolved to show interactions between stakeholders and perceived risks/opportunities in relation to the production, processing, sale and consumption of biofortified wheat. An infographic will be developed to illustrate these findings, and this will be published on our project website (www.uclan.ac.uk/research/explore/projects/bizifed-project.php) in 2019.

After the RCT was completed, semi‐structured interviews were conducted in May 2018 to assess awareness and acceptance of biofortified flour. A subsample of 10 (out of 50) households from the RCT was randomly selected. Five male heads of household and five female trial participants were interviewed. The interview schedule was developed collaboratively with the field team. Questions related to participants’ experiences of using the new flour during the trial, comparisons with their usual flour, awareness of potential health benefits, willingness to purchase more of the flour if it becomes available in the future and wider community perceptions. At the time of submission of this manuscript in November 2018, interview data were being translated into English and transcribed. Thematic analysis will be completed in early 2019.

A questionnaire survey was conducted in July 2018 to explore farmers’ views and perspectives on biofortified wheat. Sixty‐six farmers from Sindh Province were recruited, who had grown the new variety of wheat (Zincol‐2016/NR‐421). Questions related to farmers’ awareness of biofortification and their experiences of growing biofortified wheat, including fertiliser use, support/advice received, and any differences in yield and price. These findings will contribute to future work around scaling up biofortified wheat in Pakistan.

## Capacity building and development of long‐term research partnerships in Pakistan

Objective 4 of the *BiZiFED* project is to establish long‐term research partnerships to develop local capacity, infrastructure and expertise (in Pakistan) for further research into sustainable and culturally sensitive solutions to micronutrient deficiencies. We are fortunate to have long‐standing partnerships with KMU and the Abaseen Foundation, who were instrumental to the success of the *BiZiFED* project. We developed these partnerships through a range of capacity building activities.

In preparation for the RCT, researchers and technicians from KMU were trained in a variety of techniques to minimise contamination and protect the integrity of the samples. We jointly developed practical documents to operationalise the study protocol (Lowe *et al*. 2018) such as data collection sheets, sample recording sheets and quality assurance procedures. Dr Jaffar Khan (Assistant Professor, KMU) visited two specialist laboratories in the US in May 2017. He received training on techniques to measure two novel biomarkers of zinc status: DNA fragmentation (using the comet assay) and dark adaptometry, which he disseminated to other members of his research team at KMU. A female nutritionist was recruited and trained to collect dietary intake data using the 24‐hour recall method. Her presence during the trial helped to increase compliance and retention for cultural reasons. She also collected the hair samples (in a private room) because the women keep their hair covered, and it would not be appropriate for a male researcher to be present. Interview skills training was provided during our mid‐project meeting in March 2018.

Our partners in Pakistan have contributed to international dissemination activities. We hosted a research symposium at the National Agricultural Research Centre in Islamabad, Pakistan, in March 2018. The whole project team participated in this event, which was attended by over 40 delegates from organisations including the British Council, Nutrition International, World Vision, Scaling Up Nutrition (SUN), HarvestPlus and the UK Department for International Development (DFID). M. Zia presented at the Agriculture, Nutrition and Health Academy Week in Accra, Ghana in June 2018. M. Zaman presented at the Nutrition Society Summer Conference in Leeds, UK in July 2018. Further dissemination activities are planned for 2019.

## Conclusion

This research will contribute to the evidence base for the potential impact of biofortification to alleviate zinc deficiency among the poorest communities. Our findings will be directly relevant to Pakistan and more widely applicable to other LMIC, particularly those where wheat is a dietary staple. This evidence will be used to inform policy and leverage impact at national and international levels. The *BiZiFED* project team is well positioned to exchange knowledge with the health sector, the agriculture sector, international NGOs, the Pakistan government and local communities. We look forward to sharing our findings in 2019.

## Funding

This work is supported by BBSRC Global Challenges Research Fund, Foundation Awards for Global Agriculture and Food Systems Research (BB/P02338X/1).

## Conflicts of interest

The authors have no conflicts of interest to declare.
